# Prospect of Internet of Medical Things: A Review on Security Requirements and Solutions

**DOI:** 10.3390/s22155517

**Published:** 2022-07-24

**Authors:** Pintu Kumar Sadhu, Venkata P. Yanambaka, Ahmed Abdelgawad, Kumar Yelamarthi

**Affiliations:** 1 College of Science and Engineering, Central Michigan University, Mount Pleasant, MI 48858, USA; sadhu1pk@cmich.edu (P.K.S.); yanam1v@cmich.edu (V.P.Y.); abdel1a@cmich.edu (A.A.); 2 College of Engineering, Tennessee Technological University, Cookeville, TN 38505, USA

**Keywords:** Internet of Medical Things, security and privacy, encryption, physical unclonable function, blockchain, named data networking

## Abstract

With the widespread and increasing use of Internet-of-Things (IoT) devices in all aspects of daily life, a hopeful future for people, data, and processes is emerging. Extensive spans allow for an integrated life cycle to be created from home to enterprise. The Internet of Medical Things (IoMT) forms a flourishing surface that incorporates the sensitive information of human life being sent to doctors or hospitals. These open an enormous space for hackers to utilize flaws of the IoMT network to make a profit. This creates a demand for standardizing regulations and a secure system. Though many authorities are making standards, there are some lacking in the system which makes the product vulnerable. Although many established mechanisms are present for the IoT network, there are a number of obstacles preventing its general implementation in the IoMT network. One of the adoption challenges is the IoMT devices itself, because many IoMT networks consist of battery-powered devices with constrained processing capability. A general overview of the different security integrations with IoT applications has been presented in several papers. Therefore, this paper aims to provide an overview of the IoMT ecosystem, regulations, challenges of standards, security mechanisms using cryptographic solutions, physical unclonable functions (PUF)-based solutions, blockchain, and named data networking (NDN) as well, with pros and cons.

## 1. Introduction

With the advancement of technology, wireless devices, wireless communication, and devices such as the Internet of Things (IoT) have managed to imprint in our daily lives. IoT is becoming a part of the internet revolution. Technology is making the possibility of bringing physical devices to the digital realm reachable [1]. IoT systems are impacting different aspects, from home monitoring to health monitoring, of people’s daily life. IoT is a network where physical devices are interconnected and it uses wireless communication technology as the backbone of the connectivity to collect and exchange data using “Things” such as smart healthcare devices [2]. IoT combines the power of data processing and analytics and it brings out the potential of the internet to make decisions for physical objects of the real world. The term “Internet of Things” is also known as “Internet of Objects”. IoT devices are electrical or electronic devices of varying sizes and capabilities that have the ability to connect to the internet. IoT devices can be applied in the home, education sector, industry, health sector, power sector, environment, communication, etc. [3].

**Figure 1:**
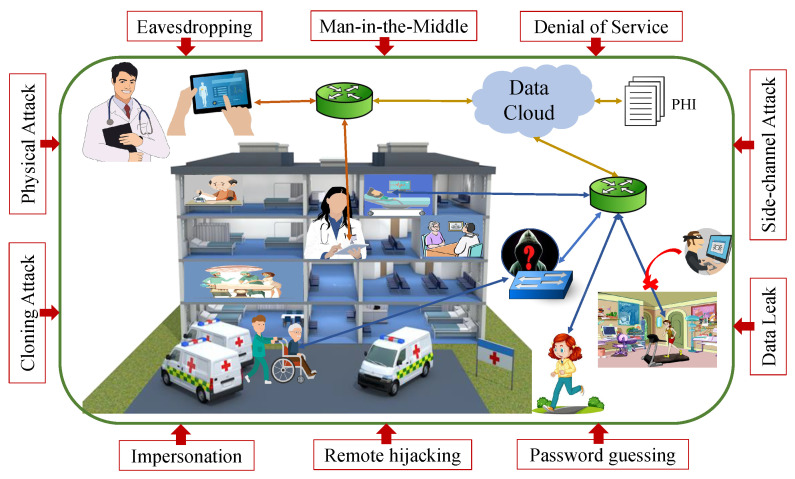
Security threats to an IoMT environment.

The growing connectivity of medical equipment has obvious implications for healthcare, including improved chronic-disease diagnosis and management for an aging global population [4]. According to a Deloitte report, more than 500,000 medical technologies are currently available [5]. IoMT devices could be wearable and medical/vital monitors; strictly, they are intended to be used on bodies, in the household, community, clinic, or healthcare facilities for medical treatment; and they may include real-time location, telemedicine, and other services [6]. The World Health Organization (WHO) defines e-health or IoMT as the application of information and communication technology (ICT) in the field of health. Electronic health records (EHR), patient health records (PHR), and mobile health are examples of subdomains within the e-health industry (m-Health). The Internet of Things (IoT) for health is widely used in e-Health areas [7]. Figure 1 shows the security vulnerabilities of the IoMT environment. The environment consists of various healthcare instruments used to gather data; an Internet connection method; and the tool/software used to process, secure, send, and visualize information [8]. Both wearable and implantable sensors collect data on the human body, send the data to a cloud server through the internet or using gateway, and the cloud server stores the data as a patient health information (PHI) file for further accessing by doctors/clinics [6,9]. The IoMT industry is equipped with smart products, such as wearables and clinical monitors, exclusively for use in healthcare settings such as the residential, community, clinic, or hospitals; as well as related telehealth services; other applications; and real-time tracking [6]. An IoMT environment is broadly classified into five segments, as discussed below:

### 1.1. On-Body Segment

Consumer health wearables and clinical-grade wearable technology are included in the on-body segment medical devices (MDs). Measurements of muscle forces, joint torques, muscle co-activation, etc., are examples of in-body data. These data are essential for understanding musculoskeletal illnesses, enhancing the effectiveness of clinical evaluations of them, carrying out duties related to health monitoring, and enhancing therapeutic treatment [10].

- Consumer health wearables: MDs that are used for a healthy lifestyle or fitness (e.g., activity trackers, fitness bands, wristbands, smartwatches sports watches, smart shoes, and clothes) are considered consumer health wearables. Companies such as Misfit (Fossil group), Garmin, FitBit, Ava, Withings, Owlet, Samsung Medical, and Apple are operating in this space.
- Clinical-grade wearables: MDs such as a smart belt for detecting falls and providing hip safety for elderly wearers. During physical activity, the Halo Sport headset is used to stimulate brain regions associated with muscle memory, stamina, and endurance. In addition, for identifying sensory nerves and proving relief from chronic pain, a wearable neuromodulation device such as Neurometrix’s Quell is used. Medical-grade devices include any wearable endorsed by one or more health regulators and authorities such as the U.S. Food and Drug Administration (FDA), whereas there is no involvement of health authorities in the segment of consumer health wearables.

### 1.2. In-Home Segment

In this segment, the home determines the residents’ health statuses and behavioral patterns based on sensor information. The house provides residents with mobile devices, display systems, and home robots while automatically controlling intelligent devices [11]. This segment is divided into personal emergency response systems (PERS), remote patient monitoring (RPM), and telehealth virtual visits (TVV). Chronic-disease management, in-home elderly care, and remote drug management are all possible with these technologies.

- PERS: These MDs are used for senior or dependent members to avoid mobility. This combines wearable devices/relay units with a live medical call-center service to provide prompt medical assistance.
- RPM: It monitors physiological parameters continuously in an effort to slow disease progression, reduce healing time, and avoid re-hospitalization. It comprises all home-monitoring devices and sensors that provide medication reminders and dosing information.
- TVV: Telemedicine and digital tests are an example of TVV. It helps patients obtain treatment and prescriptions or recommended care plans without a hospital visit.

### 1.3. Community Segment

The community segment [12] considers cities’ or areas’ MDs and stations. This segment consists of five components:

- Mobility: this service keeps track of health parameters for patients in transit.
- Emergency response intelligence: this is designed to assist primary patients such as first responders, nurses, and hospital emergency-department care providers.
- Kiosks: this includes things such as computer touchscreen displays that may dispense products or provide services such as access to healthcare providers.
- Point-of-care devices: MDs that give services outside of the home or in health-care settings such as medical camps.
- Logistics: devices such as sensors in pharmaceutical shipments that measure pressure, temperature, humidity, shock, and tilt.

### 1.4. In-Clinic Segment

To perform the operations of clinics, MDs help to collect information and provide suggestions [6].

- MDs that perform administrative or clinical functionalities.
- MDs who are employed in administrative or clinical roles.
- A service provider can be situated in another location while a product is being used by qualified personnel in this segment.

### 1.5. In-Hospital Segment

This category makes up IoMT-enabled devices and a larger number of solutions in several management areas such as asset management, personnel management, patient flow management, inventory management, environment (e.g., temperature, pressure, and humidity), and energy monitoring in hospitals. Devices such as Zoll’s wearable defibrillator and Stanley Healthcare’s hand-hygiene compliance system are included in this segment [13].

Eavesdropping, data leak, denial of service (DoS), physical attacks, cloning, side-channel attacks [14], remote hijacking, impersonation, password guessing, and man-in-the-middle (MITM) [15] are some examples of potential hostile threats. In the context of a physical attack, the attacker needs to be in close proximity to the device. If the intruder can capture the device, by obtaining some sensitive information and duplicating the device, a cloning attack can be performed [16]. Timing and power analysis are used in cases of a side-channel attack. A hacker intercepts, removes, or alters data being transmitted between two devices in an eavesdropping attack. In order to obtain the private data of two IoT devices, an attacker is able to listen to or observe their communication in the MITM. In a DoS attack, service is interrupted by blocking resources [17]. To avoid attacks, the IoMT network needs to be secured.

**Figure 2:**
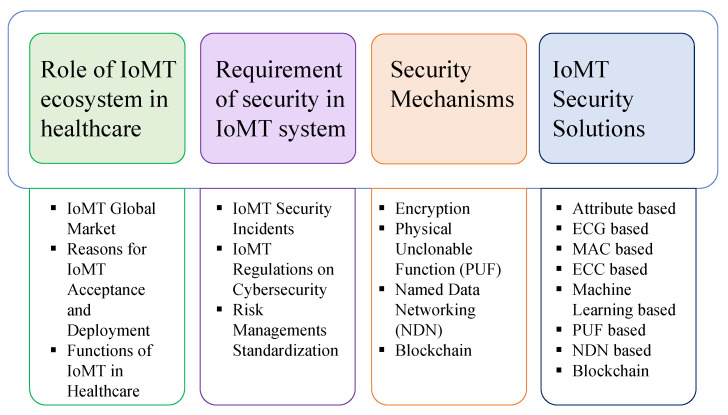
Paper organization.

It is evident from the above that IoMT is becoming a vital part of human life and it is required to create a trusted environment to operate the technology and preserve confidential information. The rest of the paper is organized as in Figure 2.

## 2. Contributions

IoMT is a critical system as it is related with human life and confidential information. To make the system secure, different types of experiments are ongoing. It is attempted in this survey paper to provide a complete and organized view of the system and recent research outcomes to provide understanding of the field in this work. There are many researchers who have provided good overviews in the arena of IoMT. Shamsoshoara et al. [18] provided security measures using PUF; Fernández-Caramés et al. [19] showed the challenges of security measures; Alwarafy et al. [20] presented intrusion detection using edge computing; Shakeel et al. [21] showed security systems in limited scale; Al-Garadi et al. [22], Arora et al. [23], and Rbah et al. [24] presented deep-learning based intrusion detection system; and only blockchain-based measures are presented by Sengupta et al. [17], Sawadi et al. [25], and Khor et al. [26]. This paper presents not only the issues and challenges of IoMT application that are highlighted, but security and privacy risks with both centralized and decentralized solutions are presented. The key contributions of this article can be summarized as below:

- Overview of IoMT network, IoMT device segments, and threats to the network;
- The importance of the IoMT ecosystem and role played in the current world is highlighted;
- A review of regulations related to healthcare devices is presented and issues are listed;
- Security mechanisms that are widely used for resource-constrained devices are discussed;
- The pros and cons of the different proposed security frameworks are provided; centralized, decentralized and NDN, as well, are presented.

Table 1 lists the goals of several survey papers and describes how this paper’s perspective differs from that of other review papers.

## 3. Role of IoMT Ecosystem in Healthcare

Day by day, the usage of IoMT is rising. In the COVID-19 situation, the adaptation of IoMT has been boosted more than in previous times due to the promising features of the ecosystem. In this section, the role of the IoMT system and the growth in the global IoMT market will be presented.

### 3.1. IoMT Global Market

**Figure 3:**
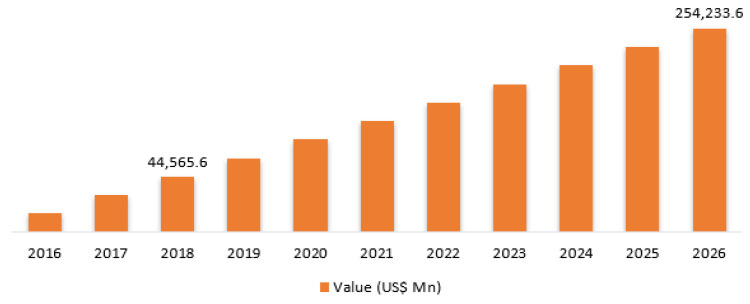
Global Internet of Medical Things(IoMT) market, 2016–2026, USD Mn [28].

In a report of UnivDatos Market Insights, the Global IoMT market is expected to grow at a compound annual growth rate (CAGR) of 18.5% from 2021-2027 to reach USD 284.5 billion by 2027. It was found that, as of 2020, connected MDs accounted for around 48% of the total MDs and, by 2025, the share would increase to around 68%. It was revealed that there are around 50,000 medical technologies that are available as of 2020. In addition, it is anticipated that IoMT technology would save around USD 300 billion in expenses annually in the healthcare sector. As of 2020, the R&D spending to connected MDs is around 34%, which will reach 42% by 2025. Another report by AllTheResearch [28], in Figure 3, shows that, by 2026, the IoMT market is estimated to reach USD 254.2 billion, with a CAGR of 24.4%. According to AllTheResearch, this is a significant rise from USD 44.5 billion forecasted in 2018, and the smart-wearable-devices segment is expected to dominate the market throughout the forecast period [29].

**Figure 4:**
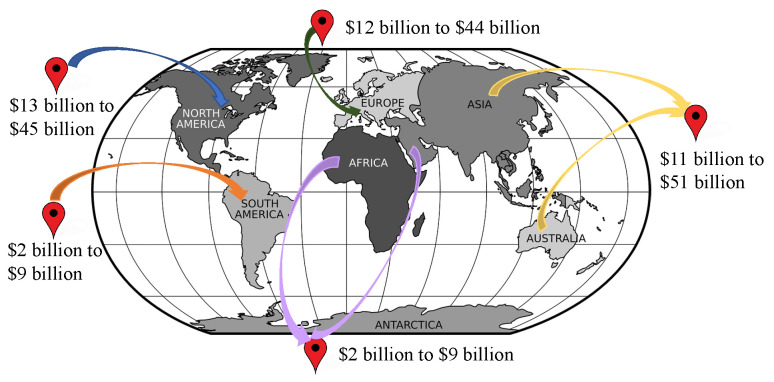
Global Internet of Medical Things (IoMT) growth prediction.

The Deloitte report has found that the IoMT global market is worth USD 158.1 billion in 2022, which was USD 41 billion in 2017. It also, further, states that the industry allocates a budget of 34% for R&D now, which will be increased to 42% in the next 5 years [30]. The estimated growth in healthcare provision is from USD 7.1 trillion in 2015 to USD 8.47 trillion by 2020 and it will increase rapidly, as by 2040 the number of elderly people will have doubled [5]. It is evident that IoMT devices are becoming an integrated part of the modern health system and the future’s medical system will be fully dependent on IoMT to reduce costs, time and improve the healthcare system. Figure 4 shows the global IoMT growth prediction in 2022.

### 3.2. Reasons for IoMT Acceptance and Deployment

**Figure 5:**
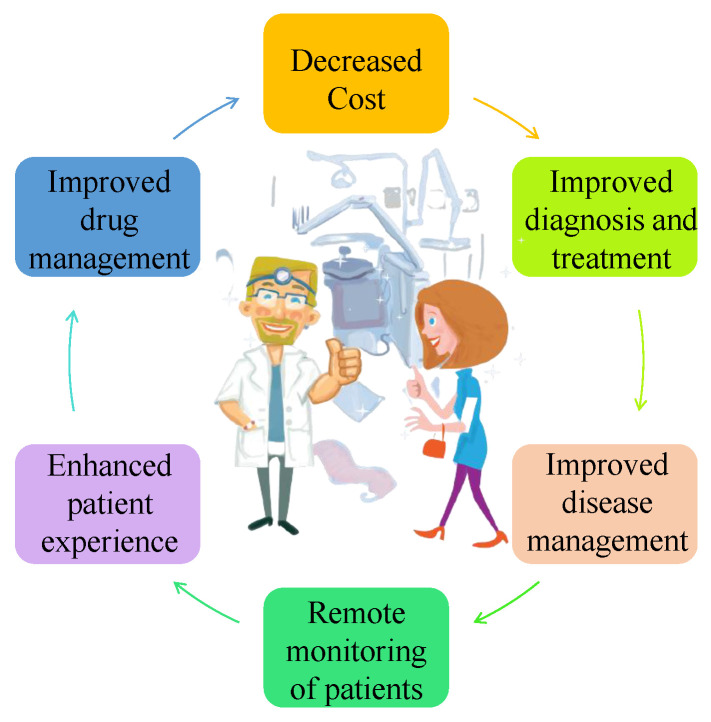
Benefits of IoMT systems.

The Deloitte report [30] states that a reduction in healthcare costs, and the opportunity of remote patient monitoring, improved patient treatment, and satisfaction are the key enabler of IoMT embracement, as shown in Figure 5.

Gus Vlahos, the Director of Healthcare Sales for CDW in the Central Region, mentions five reasons for the acceptance of IoMT devices in healthcare organizations [31].

- Boost and speed up clinician workflows: Small and compact tools, such as Memorial Hermann Health System in Texas, offer significant convenience at the point of care. These tools are used for performing critical duties such as sending SMS, scanning barcodes, and transmitting images.
- Empowers extreme connectivity: Advanced automation and perceptions are at the core of MDs functionality. For instance, smart pills and ultrasound machines can send real-time notifications or alert messages to healthcare organizations.
- Remote medical care and services: It allows patients–doctors communication and monitoring from distances. Wi-Fi or Bluetooth-enabled devices such as blood pressure cuffs or glucose monitors or heart-rate checkers collect and transmit data to doctors for evaluating patients’ conditions and prescribing accordingly. For example, improved medication adherence and lowered costs have been achieved by remote patient monitoring (RPM) in different hospitals across Europe such as UCLA Health and Children’s Health in Dallas.
- Proactive approach for sustaining good health: The widespread use of consumer wearable devices allows to aggregate and send patients’ health data to medical personnel for required investigation and treatment. Instead of reactive treatment, it provides proactive care. Wearable devices such as smartwatches and fitness bands are on the market with new types of sensors to keep track of heart rate, measure blood oxygen levels, and provide alerts by forwarding messages.
- Prioritizing the importance of effective security measures: Despite their many benefits, MDs face security issues as a result of device mobility and network requirements, unresolved default passwords, and infrequent (if any) software upgrades. A greater emphasis should be placed on end-to-end security techniques and reliable network monitoring.

### 3.3. Functions of IoMT in Healthcare

The IoMT helps to reduce the operating cost of healthcare organizations, assists in the precise identification of diseases, lowers error rates, and allows patients to communicate directly with doctors remotely. According to a Deloitte report, IoMT has lead to USD 2.5 million in savings in one year, a 90% reduction in patient admission time, 33% reduction in the length of stay for cardiac-resynchronization therapy, 37% reduction in canceled procedures through better planning and scheduling patients, and a 43% reduction in staff overtime. There is significant importance in the current COVID-19 crisis; it has been used in conjunction with other techniques to stop COVID-19 from spreading. It builds a shield for the safety of front-line personnel, improves efficiency which reduces the disease’s impact on people’s lives, and eventually lowers the mortality rate. IoT’s scalability allows for remote supervision of a significant proportion of patients from their residences or hospitals, with no need for an in-person visit. AllTheResearch shows that IoMT deployment is further accelerated due to the global pandemic of COVID-19 and is playing a vital part in the advancement of the technology. Wireless sensor network (WSN), Bluetooth, ZigBee, WiFi, NB-IoT, LTE, 4G, and 5G along with big data, artificial intelligence, and cloud computing are playing a significant role and making a competent health-tech ecosystem [32].

## 4. Requirement of Security in IoMT System

With a widespread adaptation of IoMT, the data handled by the environment has become more vulnerable. Sensitive user data will be at risk if an attacker gains access to the environment with an ill intention; in certain situations, patient life might be at risk. Many such vulnerabilities have been found through research and community support. This section presents some of these vulnerabilities of IoMT environments and the regulations in place to defend against them.

### 4.1. IoMT Security Incidents

Security and privacy (SNP) are the most critical characteristics to achieve in a healthcare environment for providing trust and high-quality services. Although SNP is a major concern in all other systems, SNP is fundamentally different from other systems, as patients’ health and life are dependent on the healthcare system. The security of computing-systems targets the CIA triad by protecting and safeguarding hardware, software, and information: (1) confidentiality: secrecy of information or resources; (2) integrity: prevention of unauthorized access to data and maintaining trustworthiness to protect data resources; (3) availability: accessibility of data whenever required. Malicious attackers are developing new tactics and techniques to penetrate security within enterprises, resulting in data theft, tampering, or blackmailing, for example. IoMT has suffered from several security incidents. There were approximately 115 reported cyber-attacks in January of 2018. A breach caused the most damage to Health South-East RHF, a medical organization that oversees hospitals in Norway, with over 2.9 million subscribers adversely impacted. One of the most significant and critical medical breaches, the WannaCry ransomware hit on England’s National Health Service (NHS), resulted in the cancellation of 19,000 appointments, and expenditure of GBP 92 million was required to mitigate and recover from the disaster [33].

**Figure 6:**
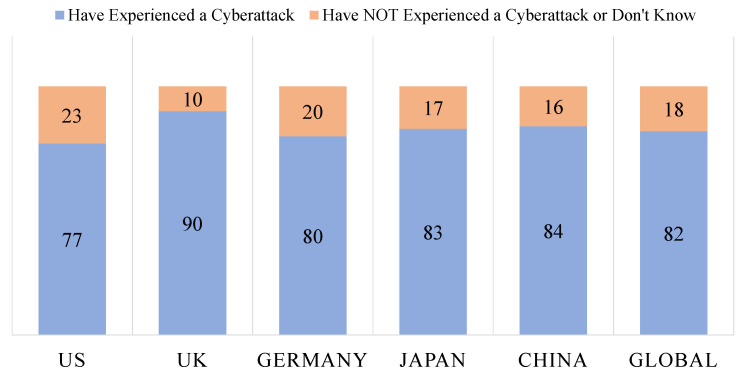
Irdeto 2019 survey results.

About 90% of healthcare organizations that have deployed IoMT have suffered at least one security breach. Another study found that 35% of organizations, more than 370 organizations using the IoMT, faced at least one cybersecurity breach in 2016. In reality, in 2017, IoMT suffered 45% of total ransomware attacks. MEDJACK 2 showed the successful instantiation of ransomware attacks in IoMT environments and this process caused the stealing of data. In 2017, more than 200,000 devices worldwide were affected by the largest ransomware attack [34]. In December 2020, the Department of Homeland Security’s Cybersecurity and Infrastructure Security Agency issued an alert to all hospitals and other medical delivery organizations about an authentication vulnerability discovered in multiple GE Healthcare machines by healthcare cybersecurity vendor CyberMDX, which poses a serious risk to protected health information. It has a critical severity rating of 9.8 out of 10. Another warning was issued regarding several MedTronic MyCareLink (MCL) MDs that may have an effect on clinical data. The vulnerabilities were discovered in all editions of the MCL Smart Model 25000 Patient Reader, with an 8.8 out of 10 severity rating. According to research published by Forescout Research Labs, a total of 33 vulnerabilities were discovered in four open-source TCP/IP stacks, affecting over 150 vendors and millions of MDs. DNS, IPv6, and TCP components are all affected by the AMNESIA:33 issues, which are linked to Ripple20 [35]. A hospital in Indiana had to pay USD 50,000 to restore its data in 2018. Figure 6 shows the survey result of Irdeto 2019 regarding MD manufacturers who were asked whether they received at least one report of a cyberattack on their products in the 12 months preceding the survey [36].

### 4.2. IoMT Regulations on Cybersecurity

**Figure 7:**
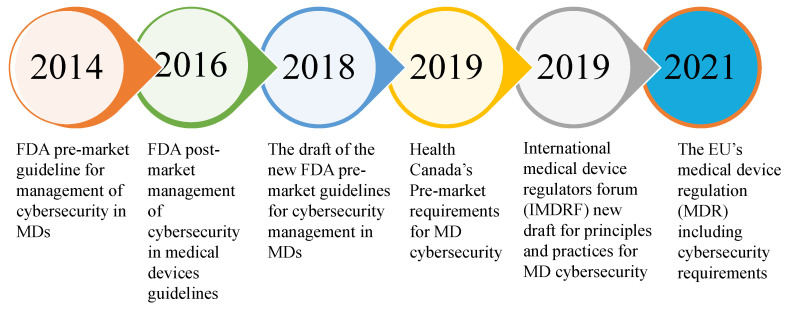
Evolution of medical device cybersecurity regulations.

To address, manage, and lessen safety risks for users and patients, major governmental bodies are working to update their pre-market cybersecurity requirements for MDs. The Food and Drug Administration (FDA), the government agency that supervises and regulates the MD industry, keeps an eye on the security incidents of MDs to improve the IMDs security and ensure that patients are safe. As a first step, the FDA released guidance on the Premarket Submissions for Management of Cybersecurity in October 2014. Using these guidelines, recommendations were made for improved security management and risk reduction so that device operations would not be affected, either purposefully or unintentionally. It is requested to all the manufacturers to follow the recommendations during the design and development phase of MDs to guard against cybersecurity vulnerabilities. A cybersecurity vulnerability and management approach establishment was also encouraged as part of their software validation and risk analysis. In 2016, the draft guidance of postmarket management recommended implementing an effective cybersecurity risk-management program for both premarket and postmarket lifecycle phases [37]. In 2018, a draft of new pre-market guidance was released to put forward a novel category for Tier 1 and Tier 2 connected MDs based on their connectivity and the scale of the harm they potentially carry if seized. Based on that demonstration, 501K Tier 1 MDs should be analysed to ensure that they follow FDA requirements regarding the design and risk assessment activities. Figure 7 shows the evolution of MD cybersecurity regulations [36].

As a regulation body, the FDA encourages medical manufacturers to design “Trustworthy” devices. According to the FDA, trustworthy devices:

- Are reasonably secure from cybersecurity invasion and compromise.
- Provide a standard level of availability, reliability, and correct operation.
- Are reasonably suited to performing their desired functions.
- Adhere to generally accepted security procedures throughout.

Other than this, it recommends a framework similar to the standardization body NIST, proposes new labeling instructions, and requests companies to share a Cybersecurity Bill of Materials (CBOM) with customers. NIST followed security vulnerabilities and provided a revised checklist of SNP controls (NIST 800-$53^{2}$) and the Privacy Engineering Program (PEP${}^{3}$), and the ISO’s standard on privacy engineering for system life-cycle processes (ISO/IEC TR 27550:2019${}^{4}$) [38]. In the domain of mHealth and uHealth systems, it is essential to achieve compliance and implement standards. Due to the COVID-19 pandemic, the EU MDR cybersecurity requirement has been postponed to 2021. It sets up a “defense-in-depth strategy”, a framework of requirements to ensure devices are secured.

### 4.3. Risk-Management Standardization

To make the MDs as error-free and secure against adversary, both FDA and EU are consistently working and making standardization to create an ideal healthcare ecosystem. Here, a few of the standardization frameworks will be discussed.

#### 4.3.1. ISO 14971 Medical Devices—Application of Risk Management

**Figure 8:**
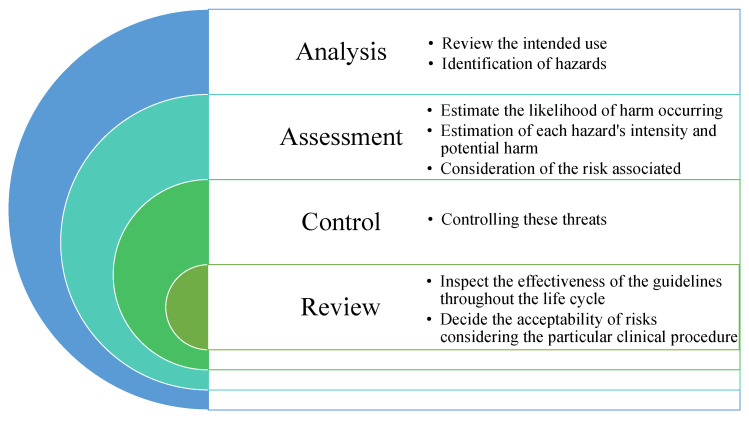
Risk-management process of ISO 14971.

This is a standard of nine parts that first specifies a process of risk analysis, assessment, control, and inspection, as well as a system for review and monitoring throughout and after production [39]. Though the standard is primarily applicable for the risk management of MDs, the framework can be used for other products [40]. The European Directives related to MDs (Active Implantable Medical Device Directive (AIMDD), In-vitro Diagnostic Medical Device Directive(IVDD), Medical Devices Directive(MDD)) are standardized in EN ISO 14971:2012 through the three “Zed Annexes” (ZA, ZB, and ZC). Through Annex ZA, the MDD 93/42/EEC of 1993 was standardized with ISO 14971:2012. Annex ZB standardized the AIMDD 90/385/EEC of 1990. Annex ZC harmonized the IVDD 98/79/EC of 1998 [39]. The latest standard is ISO 14791:2019, which was approved by the Association for the Advancement of Medical Instrumentation (AAMI) and by the American National Standards Institute (ANSI) on May 2, 2019 and May 10, 2019, respectively. ISO 14971:2019 specifies the guidelines for risk-management systems of MDs, as well as practice standards for a device’s whole life cycle. Figure 8 shows the ISO 14071 risk-management procedure [41,42].

Risk analysis is a holistic approach that uses all available data to determine the dangers associated with MDs and assess the potential consequences. During the risk-evaluation phase, the manufacturer must assess the projected risks for every identified hazardous circumstance and determine if the risk is within the accepted range or not using the acceptability criteria of the risk-management plan. If the risk is deemed acceptable, it is classified as a residual risk under the standard. If not, risk security controls for each dangerous circumstance must be carried out to lower the risk to an acceptable level. The findings of the risk assessment should be kept in the risk-management file [43]. To limit the approximate risk, the manufacturer needs to design safely, take protective measures during production to the process or device itself, and prepare safety guideline for users. The devices need to be reviewed both pre- and post-manufacturing [41]. Instructions for Use (IFU) suggested single fault criteria are not followed [44]. In [45], Hatcliff et al. identified the following challenges in distributed risk management to adhere to ISO 14971.

- Defining the scope, bounds of risk management, and intended use.
- Need for proper correlation between hierarchical system architectures and terms such as hazards; need for clear definition of root causes and effects.
- Avoiding robust approaches to ensure safety and security.
- Lack of “safety class” with respect to reliability and trustworthiness which exists in ISO 62304.
- Risk control while multiple items are involved; reliable risk controls.
- Verify each safety component, test items in different systems or configurations.
- Disclosures are required for development users and operational users, Balancing the necessity of external stakeholders with proper communication.

Despite not defining acceptable risk thresholds, this agreement compels manufacturers to develop objective standards for risk tolerance. The company is not, however, required by this agreement to have a quality-management system in place [46]. Device safety issues can be calculated probabilistically based on historical data or modeling. The FDA stated that, while cybersecurity breaches do not follow a probabilistic model, equipment safety issues can and that this difference is not taken into consideration in the ISO standard [46].

#### 4.3.2. ISO 13485 Medical Devices—Quality Management System (QMS)

**Figure 9:**
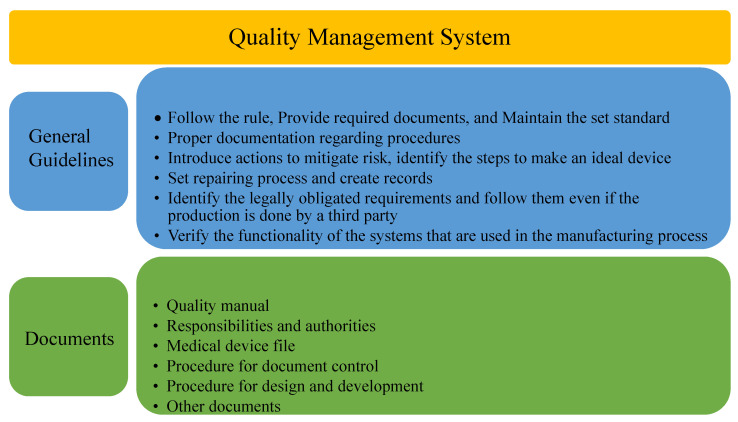
Quality management system of ISO 13485.

ISO 13485 gives manufacturers a realistic foundation for addressing the MDD, the EU Medical Device Regulation (MDR), and other laws, as well as exhibiting a focus on MD safety and reliability [47]. ISO 13485 was first published in 1996, then it was modified in 2003, 2009, 2012, 2016. The latest version was published on 9 September 2021 [48]. It focuses on risk, defines responsibilities of management, clarifies training responsibilities, shows the improvement scope of facility management, provides better design alignment, supports many development regulations, emphasises supply control, supports traceability procedures, and defines complaint-handling processes and product-cleanliness requirements. It is divided into eight sections, where the first three sections are introductory and remaining five are mandatory. The fourth section is QMS, which is further divided into general requirements and documentation requirements. Figure 9 shows the QMS requirements [47]. The fifth section is about managements’ responsibility to demonstrate their dedication by demonstrating that they can be made responsible for their company’s activities. Resource management, in the sixth section, focuses on adequate resource management to maintain quality and deliver promised devices. The seventh section is about product realization to make a proper project plan to implement the device concept. The last section is measurement, analysis and improvement in dealing with customers, regulations, and future improvement, etc. [47,49].

Though ISO 13485 is not mandatory for maintaining compliance, it is advisable to follow the standard to ensure design, development, production, quality, and delivery [48]. Companies from EU, Canada, Australia, and Japan are committed to following ISO 13485 and all 165 member countries follow the framework as well [49]. From 2012 to 2018, each year, around 30,000 organizations received certificates [47]. Furthermore, the FDA proposed a rule that will harmonize ISO 13485 criteria [49,50]. As the ISO 13485 is being accepted by companies, the cybersecurity scopes should be distinguished in detail. In the section “4.2.5 Control of records”, it only states that, in order to specify the controls required for the identification, storage, security and integrity, retrieval, retention period, and disposal of documents, the organization must document procedures. The section is more focused on records rather than security-measure clarification [51].

#### 4.3.3. IEC 60601-1—Medical Electrical Equipment

The International Electrotechnical Commission (IEC) has published a set of specifications for the safety and required quality of medical electrical systems known as IEC 60601, which was first published in 1977. It focuses on building electric medical equipment no matter whether it is being used by a layperson or trained medical professionals in a home-healthcare environment [52]. The environment could be a patient’s living place or another place where individuals can be found both inside and outside. The standard focuses on home medical systems as every person will not understand the risks associated with electricity exposure. It can cause devastating situations; for example, electric currents as low as 50 mA can flow through a human’s skin, causing fibrillation in the heart and paralysis of the breathing system [53]. To avoid this kind of circumstance, the IEC developed standard 60601, which consists of two parts: IEC 60601-1 and IEC 60601-2. The IEC 60601-1 3rd edition is a set of standards for medical electrical equipment that serves as a foundation for evaluating their efficacy and safety. The following features are required to be considered while developing equipment:

- Sharp edges.
- Pinch points.
- Stability under various conditions.
- Movement over a threshold.
- Rough handling.
- Strain on the user.
- Durability of marking.
- Tensile safety factor.
- Correct labeling.

The standard has been adopted all over world especially the US, Canada, EU, Japan, Australia, Brazil, etc. However, due to a lack of understanding and no explicit regulated risk-assessment techniques in existence, many companies are hesitating to adopt [54]. The intricacy, cost, and commercial risk associated with the 60601 certification procedure have all been questioned. The uncertain adoption date of the new revision has been a particular source of concern during the migration to the third edition [55].

#### 4.3.4. ISO/IEC 80001—Risk Management of Medical Devices on a Network

This standard, sometimes referred to as 80001, emphasizes the need for collaboration between healthcare providers, MD makers, and IT vendors. It establishes the necessary functions and responsibility, as well as a procedure for controlling the risk imposed by the introduction of MDs into the public healthcare organization’s IT infrastructure [56]. This standard is not applicable for applications used by individuals. The standard is divided into two parts [57]:

- ISO/IEC 80001 [58]: Application of risk management for IT-networks incorporating medical devices—Part 1: Roles, responsibilities, and activities in ten sub-parts, dealing with risk-management techniques, guidelines, and processes.
- ISO/IEC 80001: Application of risk management for IT-networks incorporating medical devices—Part 2—1: Roles, responsibilities, and activities in ten sub-parts, dealing with risk-management techniques, guidelines, and processes; Practical Applications and Examples in nine subparts dealing with technical controls and requirements to enable the implementation of ISO/IEC 80001-1.

Specifically, the sub-parts that correspond to integrated security measures are ISO/IEC 80001-2-8 [59]: Application guidance—Guidance on standards for establishing the security capabilities identified in IEC 80001-2-2, which maps and translates the presented capabilities from ISO/IEC 80001-2-2. The ISO/IEC 80001 standard listed 19 security capabilities, which are automatic log-off, audit, authorization, configuration of security features, cybersecurity product upgrade, health data de-identification, data backup and recovery, emergency access, health data integrity and authenticity, malware detection and prevention, node authentication, personal authentication, physical locks and devices, third-party components in product lifecycle roadmaps, software and application hardening, security guidelines, health data storage and confidentiality, transmission confidentiality, and transmission integrity [57]. The ISO/IEC 80001 standard intends to offer a minimum degree of cybersecurity, but one study [57] shows that the standard has flaws and identifies critical aspects of cybersecurity that might be improved.

#### 4.3.5. EU MDR 2017/745

A European Union rule on the clinical testing and marketing of MDs intended for human use is known as Regulation (EU) 2017/745. The rule was released on 5 April 2017, and it became effective on 25 May 2017 [60]. After three years’ transition time, the application came into effect in 26 May 2021. Based on the sponsor type, the MDR’s 10 chapters, 123 articles, and 17 annexes cover medical evaluations and clinical trials with MDs in the EU. The MDR replaces MDD 93/42/EEC and AIMDD 90/385/EEC [61]. In article 2(1), the MDs are defined as [62]: any instrument, apparatus, appliance, software, implant, reagent, material or other article intended by the manufacturer to be used, alone or in combination, for human beings for one or more of the following specific medical purposes:

- diagnosis, prevention, monitoring, prediction, prognosis, treatment or alleviation of disease

Softwares for medical purposes were not included in the previous standard, which is listed in the MDR [62]. The MDR suggests tighter regulation and enhanced pre-market infrastructure. Additionally, the obligations for producers’ post-market monitoring as well as for the recognized bodies’ responsibilities will be expanded and strengthened. A unique device identification is required to be added to EUDAMED, which is the EU database [63]. By making all manufacturer activity publicly accessible, the EUDAMED will play a significant role in supplying the required transparency and traceability [64]. The new MDR brings a lot of improvements in many areas such as HA filler, approval process, certification, CE marking, clinical evaluation, post-market surveillance, etc. that makes the MDR as trusted to the FDA [65]. There are a few difficulties with the conditions that MDR placed on software developers that still need to be clarified. The European Commission has a responsibility to resolve and clarify those issues [63].

#### 4.3.6. FDA-2020-D-0957—Guidance for the Content of Premarket Submissions for Software Contained in Medical Devices

This guideline is applicable for those MDs that contain one or more software components, or parts. The guideline divided concerns into three levels: major, moderate, and minor [66]. If a fault or malfunction could directly cause a patient’s or an operator’s death or a severe accident, it is considered to be a major worry. The major-level concern is also applicable for blood establishment computer software and also if the software is associated with a drug or biologic. If a malfunction or a latent software bug could cause minor harm to people or workers, the level of concern is moderate. If malfunctions or latent design defects are unlikely to harm the patient or operator, the level of worry is minor. It is required to submit the device design, implementation process, testing process, efficient hazard identification, countermeasures, and traceability matrix to combine design, production, testing and threat management documents [66].

#### 4.3.7. FDA-2013-D-0616—Content of Premarket Submissions for Management of Cybersecurity in Medical Devices

In 2005, the FDA addressed the necessity of cybersecurity in the premarket stage for the MDs that incorporate software. In accordance with the Quality System Regulation (21 C.F.R. part 820), 10 fundamental questions and answers are first provided that outlined the need to take cybersecurity vulnerabilities in devices using off-the-shelf software into consideration [67]. According to the FDA-2013-D-0616, companies should establish cybersecurity-related support or guidance for their products and establish cyberattack vulnerability and trust management in the stage of software validation and risk analysis required by 21 CFR 820.30. The following components should be appropriately covered by the strategy [68,69]:

- Identification of assets, threats, and vulnerabilities.
- Evaluation of the effects of risks and attacks on device features and functions and end consumers.
- Evaluation of the propensity for a threat and a weakness to be addressed.
- Determining risk exposure levels and effective mitigation tactics.
- Evaluation of overall risk standards and residual risk.

The FDA suggested that identify, protect, detect, respond, and recover are taken into account by companies to direct their cybersecurity efforts [69], by limiting access to trusted users only and ensuring trusted content companies can fulfill identify and protect functionalities. Implementation of features to detect anomalies, train customers to identify cybersecurity events and run critical functionalities even when affected by attacks, and retention and recovery methods implementation are covered [68]. Analysis, mitigation, and design related to risk, a traceability matrix to control cybersecurity, a software update plan, a control summary to maintain integrity, and instructions to use, and defence against cybersecurity are the required documents for premarket submission [68,69]. To cater to security requirements, the NIST Cybersecurity Framework provides a mechanism to define cybersecurity operations, desired results, and relevant references that an MD maker could use to create their cybersecurity management program. Unfortunately, the FDA completely ignored this opportunity. Rather, the Guideline provides two pages of “motherhood and apple pie”-style bullet points. For instance, under the “Limit Access” area, they offer such revolutionary suggestions as using passwords, biometrics, etc. [70].

#### 4.3.8. FDA-2015-D-5105—Postmarket Management of Cybersecurity in Medical Devices

The FDA is publishing this recommendation to let the industry and FDA personnel know what the agency thinks should be carried out concerning post-market security breaches for commercialized MDs. The guideline emphasises that producers should track, detect, and remedy security vulnerabilities and attacks as part of the postmarket management of their healthcare devices and explains the FDA’s postmarket guidelines with respect to this issue of cybersecurity vulnerabilities [71]. The FDA identifies critical components as below [72]:

- Monitor to identify and detect sources of security vulnerabilities and threats.
- Preserving a robust software lifecycle that includes software update design, verification, third-party software monitoring.
- Understand, assess, and detect the presence and effect of a hazard.
- Establishing and sharing procedures for identifying and handling vulnerabilities.
- Clear definition of a threat model to maintain protection, response, and recovery.
- Establishing a unified policy and procedure for vulnerability disclosure.
- Enacting mitigations that deal with cybersecurity risk before it is exploited.

Cybersecurity vulnerabilities are divided into controlled risk of patient harm and uncontrolled risk to safety and essential performance. Manufactures need to take urgent actions if any uncontrolled risk is identified and there is a chance of severe patient loss if exploited [73]. Though the standard is placed to make the healthcare ecosystem secure, there are some unclear statements. For example, the FDA did not set the boundary of essential clinical performance and it depends on the manufactures. The scope of critical impact is not properly addressed. The standard needs to address off-the-shelf software to provide a patch when there is any security vulnerability [74].

Both the FDA and EU MDR prioritize incorporating and implementing a cybersecurity mindset from the very first steps of the product lifecycle to the end of support. IoMT is a considerably new technology, and the constraints of SNP are not yet entirely met. When the FDA-2013-D-0616 was drafted, then the Telecommunications Industry Association opposed modifying the already-submitted medical software for premarket approval for strengthening cybersecurity [75]. It is impossible to entirely minimize risks using premarket procedures alone, since cybersecurity vulnerabilities to medical equipment are constantly changing. Manufacturers must, therefore, have thorough cybersecurity risk-management procedures and paperwork that comply with the Quality System Regulation [72]. Now, it is high time to raise awareness, agree a global standard, and adopt robust security mechanisms to protect the healthcare ecosystem against cybersecurity vulnerabilities.

## 5. Security Mechanisms

**Figure 10:**
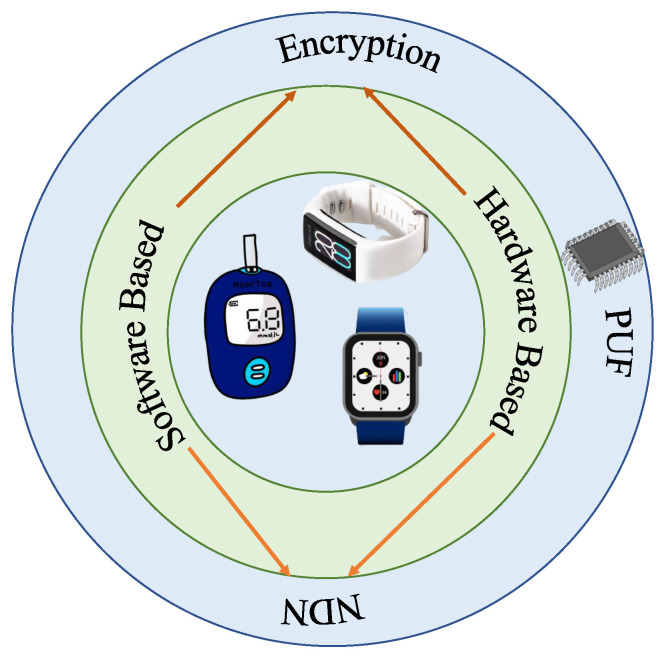
Security mechanisms.

The security mechanisms for any IoT environment are broadly categorized into two types, software-based and hardware-based security, as shown in Figure 10. In software-based security mechanisms, mathematical methods are incorporated where systems rely on software to protect the system. Though it takes time to solve mathematical approaches now, it will take less time to extract the keys when quantum computers are the reality [18].

On the other hand, in the case of hardware-based security solutions, encryption such as public key infrastructure (KPI), advanced encryption standards (AES), and elliptic curve cryptography (ECC) are being used. Pre-shared key parameters with the server or with other devices in advance are required in the case of the former ones. Though it has an advantage with respect to low computational complexity and high efficiency, it shares key parameters in the case of a high number of devices, and it is unrealistic. On the other hand, pre-shared key parameters are not required for asymmetric protocols. They utilize their public keys and private keys. Here, confidentially comes with private keys’ secrecy [76]. Device authentication and key exchange are the hurdles in achieving secure communication for IoT, in such a device-intensive environment. Furthermore, these MDs demand low computation capability, power, and storage. Many researchers are working to build an authentication system to achieve security measures. Among different authentication systems (AS), PUF is one of the hardware-based AS which requires low power and can identify the legitimate user faster. PUF provides a physically defined “digital fingerprint” that can be used as a hardware security primitive for authentication in IoMT. PUF is a physical object which generates an output (response) for a given input (challenge) that serves as a unique identifier. Due to integral physical variability in integrated circuits, it can provide a challenge–response mechanism for security applications [77]. As the name indicates, PUF is unique and cannot be cloned due to the arbitrary and intractable effects of the IC manufacturing process. Each PUF is different, and it should be able to generate random a challenge–response pair (CPR) on demand. If $C_{x}$ is a challenge input to the PUF and $R_{x}$ is the response generated from PUF, then they form a unique CRP ($C_{x}$, $R_{x}$) for a particular PUF, which can be represented as [78]

$$R_{x}=\mathrm{PUF}\;(C_{x})$$

Unlike traditional security systems, PUF does not store any password or key on the device. As there is no memory and CRPs are different for each PUF, an attacker cannot exploit PUF to use the MDs. Uniqueness, reliability, and randomness are the major characteristics of PUF [79,80].

- Uniqueness: Each challenge–response pair (CRP) of PUF is required to be different from one chip to another chip. Different PUFs should not generate the same CRP set. The ideal value of uniqueness is 50%. It is measured by hamming distance (HD). Hamming distance is measured by the difference in bits of responses.
- Reliability: A PUF should have to be reliable and have the capability to produce the same response for a particular challenge. The ideal value of the reliability of a PUF is 100%.
- Randomness: Bits (1’s and 0’s) of a response should have to be random. It is expected that, in a response, the presence of 1’s and 0’s will be the same. If a PUF follows this rule, then the randomness of the PUF will be 100%.

**Figure 11:**
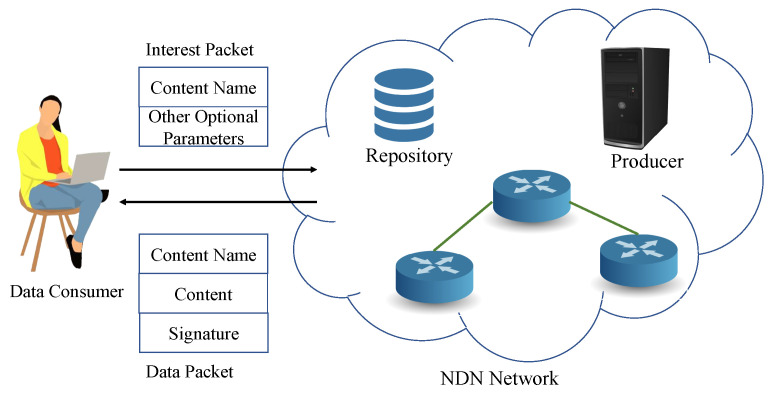
Data fetching in NDN network.

Another mechanism for protecting SNP is named data networking (NDN). It uses names to fetch data chunks. It is a proposed architecture of the internet where data communicates between sensors to servers by data bits’ names. The network layer identifies the producer by using data names of application or content. It has two types of data packets which are the interest packet (request) and data packets (response). The interest packet contains the name of the requested content, and the data packet is fetched as shown in the Figure 11. The data packet follows the same path that was followed by the interest packet [81]. Unlike the present system, the NDN router cached content to deliver to the consumer to avoid fetching data from the producer.

**Figure 12:**
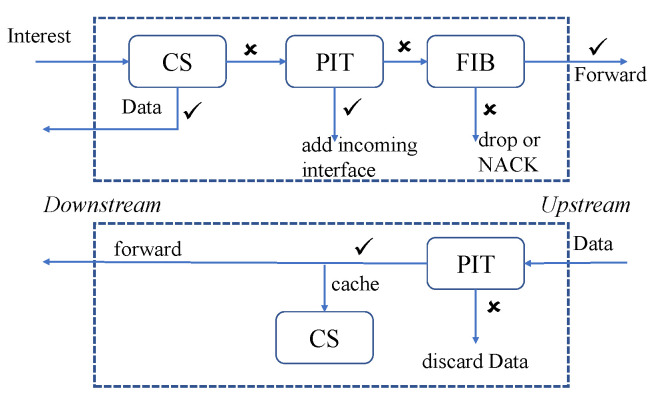
Data-forwarding process in NDN router.

As NDN packets do not contain information related to the user, it is impossible to trace who is requesting the data. Furthermore, safety is ensured as content is encrypted using a cryptographic signature [82]. In the forwarding module, there are three components which are a content store (CS), a forwarding interest base (FIB), and a pending interest table (PIT), as shown in the Figure 12. The CS caches the data. FIB manages the path to reach the destination. PIT maintains a table for each interest packet till the data packet arrives. If the data is not present in the CS, the interest packet fetches data from the producer and stores the data in the CS for future transfer.

## 6. IoMT Security Solutions

In this section, security solutions of the IoMT network based on the existing literature will be reviewed. All the schemes are presented based on the best understanding.

Chiou et al. attempted to secure the IoMT system and resist intruders trying to compromise the system by developing an authentication system in 2016 [83]. Unfortunately, Chiou et al.’s system does not provide full security to resist security threats and also does not hide the identity of patients, which was shown by Deebak et al. [84]. Though both [83] and [84] used secret keys for authentication, Ref. [83] only shared the secret key over a public channel and [84] shared the secret key by encrypting with other parameters. Though Deebak et al. improved Chiou et al.’s authentication system, it does not shield the system fully.

Park et al. [85], revealed that Xu et al. [86], are prone to impersonation, stolen senor node, and leaking verification table attacks, and their system lacks anonymity, untraceability, and reliable mutual authentication. The scheme of Xu et al. [86] stored authentication parameters as plaintext which can cause a stolen sensor node, and an attacker can log in to perform an impersonation attack using the parameters and generating nonce. Park et al. [85], solved these issues and their system does not retain the client’s authentication parameters and confidential information in the server’s database. During the registration phase, it assigns an intermediate node for communicating between sensor and server, which makes it susceptible to single point failure. It uses XOR and hash functions to make a lightweight authentication protocol. A session key will be established after verifying the intermediate node, timestamp, stored parameters, etc.

To safeguard data transfer, Kumar et al. designed an escrow-free identity-based aggregate signcryption system in [87], where a smart healthcare system collects medical data from several sensors placed on the patient’s body, signcrypts and aggregates it, and sends it to a medical cloud server through a smartphone. A network manager computes master and public keys, and issues partial private keys to an entity after authenticating. Key protection servers protect private keys and issue a protected private key and the entity extracts its private key using shares. To improve computation cost, sensors collect data along with timestamps and aggregates after signcryptexting each piece of data. The smartphone verifies the authenticity using keys and aggregates data from all sensors and sends it to the server. The doctor can decrypt the PHI file using the sensor’s private key. Though an eavesdropping attack is resisted in such a way that master key and secret keys are required to calculate the private key, it suffers from attacks such as DoS and reply.

Limaye et al. [88] showed that HERMIT, a benchmark suite, is optimizing a carefully selected set of ten emerging IoMT applications to enhance the performance of those applications and trying to expand feature for more applications. Advanced Encryption Standard (AES) and Lempel–Ziv compression (LZW) are also included to represent security and compression functions, respectively. They focus on processing IoMT data and incorporating security techniques. For this purpose, the framework includes encryption for protecting data.

Lu et al. [89] proposed a method which assumed that modules can not be tampered with to obtain a private–public key pair and if it, does the trusted server will detect this with a function. The device encrypts an index and the value of that index by a key pair and shares it with the server. The server decrypts the message using a key pair and matches the value of the index.

In [90], Hsu et al. proposed a three-factor authentication protocol using a password, smart card, and biometrics for IoMT systems. An authentication request message is transferred from a user which is composed of a function of a random number and stored shared keys. Using this information, a session key is derived during login. In the next fast authentication step, the session key is used for encryption. Though it is claimed that there is no storage for verification, it verifies the session key after defined computation. A client device stores the secret key of a particular server for authentication. The client device will face interrupted service if there is any service issue with the particular server.

Chen et al. [91], presented a chaotic-maps-based group-oriented time-bound authenticated key agreement scheme. Chaotic maps [92] have a complex nonlinear dynamical structure, which has chaotic attributes, such as large parameter space, uniform data distribution, and semigroup structure. A group key is valid for a particular time period, and a new group key will be required when it is expired as authorized entities can access within that particular period. In this work, the medical server sends a time-slot range and authentication range to both application providers and user groups. If the application provider obtains the same authentication token within the time slot, then it will complete authentication. If one member of the group is authenticated earlier, other members of the group will be authenticated using the same authentication token. How an authentication token will be shared with other members of the group is not depicted.

Li et al. [93], proposed Hash operation and XOR operation-based lightweight scheme. It has six stages and uses public channels. In the pre-deployment stage, a secure key for a sensor is shared between that sensor and the trusted gateway. This secure key helps the sensor node and user register in the gateway. Finally, the sensor and user will negotiate a session key with the help of Gateway to encrypt the data collected by the sensor node.

### 6.1. Attribute Based

Zhang et al. proposed an attribute-based encryption (ABE) authentication protocol in [94]. Here, both central authority and attribute authority are required. Users send a signed secret key and transformation key from an attribute authority to cloud user assistant and obtain a ciphertext key for mutual authentication with the cloud. ABE-based systems suffer from several problems. To begin with, the secret key does not distinguish the user, and if a fraudulent key distribution is performed, the person will not be detected. Secondly, it increases the ciphertext depending on the number of the attributes used. In addition, the required computation time is higher when users decrypt [95]. ABE cannot be applied to lightweight devices due to substantial cost during decryption [96].

Liu et al. [97], proposed a security solution where wearable devices will be connected to an edge computing server which will be in a nearby hospital. Pseudo-number generation and data attribute computation containing numbers and secret keys by client and server completes mutual authentication. Here, the secret key is divided into n segments for computation and sharing. A lot of storage and computation is required in the client device, which is not properly appropriate for IoT devices. Like Kumar et al., resistance against a few attacks such as DoS, Reply, etc., was not illustrated.

In [95], Hwang et al. proposed a ciphertext-policy attribute-based authentication (CP-ABE) protocol. In this protocol, both trusted authority and attribute authority work together to track who was issued the first key. The size of the ciphertext does not depend on the number of attributes, which causes the same decryption time for different numbers of attributes. However, the proposed scheme needs a lot of computation to verify the user’s identity. It also suffers from PHI leakage from the user who received the delegated key.

### 6.2. ECG Based

The ECG-based authentication protocol by Huang et al. [98] was developed where ECG signals are de-noised using singular value decomposition (SVD). Based on motion status and pre-defined feature templates, the noise will be reduced. A de-noised signal was achieved using weighted online SVD in case of light exercise. It is hard to obtain the angular distance for walking and running; In addition, different activities in various exercises or positions are challenging. In this work, it is considered that there is no access to patients’ ECG templates by an attacker. User identity is not properly secured, and it takes a high computation time.

### 6.3. MAC Based

Xu et al. [9] provide a MAC-based solution where a gateway collects data from MDs and stores the data in cloud servers. Data will be in an encrypted form using the pre-sharing by a trusted authority. MAC is used as an authentication system to verify the source and maintain integrity. The secure communication between IoT devices and the IoT gateway is absent. Furthermore, it is required to have a secure channel to share the computed key to the IoT gateway.

In [99], Siddiqi et al. proposed a public-key cryptography-based authentication protocol for smart cards and MDs. It is also a MAC-based authentication system. In this protocol, the server will share a hash function where the k-bit is missing, and the reader device will calculate and identify the missing k-bit. User anonymity is not in the proposed protocol.

Hahn et al. [96] showed the security vulnerability of commitment-based and MAC-based (for a value instead of a range) authentication protocol and developed a scheme where a key server will generate a verification key and commitment key. The user will use these values to compute a commitment value and share the message. A doctor will use the verification key and commitment key to decrypt the partially decrypted data to verify the commitment key.

### 6.4. ECC-Based

A two-factor protocol for WMSN was designed by Kumar et al. [100] and it is claimed to be secured against the known attacks. However, unfortunately, He et al. [101] found out that Kumar et al.’s protocol does not resist insider attacks; Li et al. [102] pointed out some vulnerabilities such as desynchronization attacks and the sensor nodes capture attack. In addition, Li et al. [103], show that it is also weak against DoS attacks. Li et al. designed an ECC-based secure three-factor authentication framework with forwarding secrecy for the WMSN fuzzy commitment technique to manage the biometric information. Meanwhile, fuzzy-verifier and honey-list techniques are used to solve the contradiction of local password verification and mobile-device lost attacks. This does not shield against man-in-the-middle attacks.

To counter Sybil attempts, Almogren et al. [104], employed a fuzzy-based trust-management technique. The trust attributes, such as integrity, receptivity, and compatibility of a node, are used by fuzzy-logic processing to evaluate trust values. Both fuzzy-logic processing and fuzzy filters are used for double evaluation checks. The introduced fuzzy filter uses two algorithms to evaluate the final trust value.

Ying et al. [105], also developed an authentication protocol using ECC and dynamic identity with user’s biometric information (ECG). During the registration period, a doctor is registered with three different random numbers, and a hashed function is burned in a smart card and provides a dynamic identity. It is not proven against server impersonation, despite its resistance to man-in-the-middle attacks. In addition, it is required to deal with ECG-related issues and has high communication costs.

Liu et al. [106], propose an attribute-based multi-keyword searchable encryption scheme. Double verification is introduced by providing the attribute-based encryption mechanism to encrypt the symmetric key and convergence key. To check the access validity of shared files, a time-division mechanism is also adopted. A user needs to generate a search token to raise an access request that the user needs to decrypt to obtain plaintext after confirming the correctness of data from the shared partial decrypted result. Here, the user-authorized cloud service provider verifies the legitimacy of the request. In this scheme, the user calculates the real private key by obtaining a private key in the registration stage. Moreover, to reduce calculations on the user side, most of the decryption calculations are performed on the cloud side.

### 6.5. Machine-Learning-Based

Wang et al. [107], proposed an effective privacy-preserving outsourced support vector machine scheme that uses eight privacy-preserving outsourced computation protocols. It uses outsourced integers and floating point computations to obtain the high efficiency and accuracy of the proposed protocol. To execute secure and privacy-preserving computation on the floating point number, it was normalized into a fix point number with fixed precision $2^{-E}$. In this scheme, an initially trusted authority distributes a public–private key pair to all participants and also extra private keys to the cloud server and provider and the becomes idle.

Awan [108] proposed an intelligent neural-network-based trust management method that evaluates the level of trust using trust factors such as compatibility, reliability, and packet delivery. It includes a smart home server that is dedicated to patients’ healthcare sensors’ trust computation and identifying malicious nodes. The smart home server receives ECC- and SHA256-based encrypted data from patients and shares it with the server after computing trust. If the server finds that the evaluated trust satisfies the threshold values, then it shares the decrypted information with the doctors. Supervised learning with an input layer, two hidden layers, and an output layer in the multi-layer perceptron neural network compute the binary output. In the first stage, it uses the summing function to add all computed parameter values, and in the second step, it uses the range limit to produce a number for trust development threshold evaluation. Decrement of trust degree can increase trust computation time and affect communication time, which makes it most challenging, as the system can identify an on–off attack.

Encryption and decryption of medical images by a deep-learning-based image encryption and decryption network are proposed by Ding et al. [109]. To complete the process of encrypting and decrypting the medical image, a deep-learning-based image encryption and decryption network (DeepEDN) is presented. To transmit from its original domain to the target domain, the cycle-generative adversarial network is used as the core learning network in this strategy. The target domain uses a reconstruction network to decrypt the image to restore the original image. In the initial convolution stage, the encryption network begins to spatially downsample and encode the images. Random keys are generated for each convolution layer encryption in the key-generation process. All the parameters of each convolutional layer are used to generate private keys for encryption. Continuously updated and refined private keys are used through the forward propagation training process of the encryption network.

### 6.6. PUF Based

It is assumed that a hardware security module or trusted authority or key generator is equipped in the previously discussed systems. The side-channel attack could affect these authentication protocols, as these required memory location/storage [110]. To avoid memory storage and resist side-channel attacks, PUF-based authentication systems are developed. One of the PUF-based security solutions for IoMT was proposed by Yanambaka et al. [111]. A hybrid-ring-oscillator-based PUF was generated for device authentication in this work. The patient device generated responses from the device based on challenges from the server and the server authenticates patients by comparing stored CRPs in the database. Though it resists client-impersonation attacks, it does not resist server-impersonation attacks.

Gope et al. [112], proposed a PUF-based authentication protocol where client devices and servers can authenticate each other and the scheme is able to resist server-impersonation attacks. In the authentication step, a client sends its pseudo-identity and nonce and the server shares a CRP pair with a generated nonce. The client verifies CRP and the server verifies the next CRP, which will be shared by the client. Usages of CRP are higher, as in each stage two CRPs are used. In addition, it reserves a few CRPs for synchronization. If reserve CRPs usages are carried out, the process of updating reserve CRPs are not mentioned. It does not completely avoid storage dependency due to reserve CRPs, and fake IDs.

Alladi et al. also developed a PUF-based authentication framework in [78] that resists man-in-the-middle attacks. Two-factor authentication is considered as authentication is required to be carried out between patient and sink node server, and sink node server to cloud server. Computations are performed using random nonce by each node, challenge, and segmented responses for a challenge. Unfortunately, it suffers from DoS attacks and complex computation. Moreover, the client device will select a challenge that should require storage for challenges. As any strong PUF does not have a sequence of CRPs, the device is not able to select challenges in a random order.

The comparative analysis of the above-discussed centralized methods are presented in Table 2.

### 6.7. Blockchain Based

Abdellatif et al. [113], propose a holistic framework that exploits the integration of edge computing and blockchain to process medical data. Initially, an automated patient-monitoring scheme was designed and, in this blockchain system, three different channels are considered for segregating different kinds of data such as urgent data. To provide minimum latency, urgent data was given the maximum priority and will deal with a less-restricted blockchain, i.e., with a minimal number of validators by supporting swift response. This work mainly focuses on priority assignment and feature extraction for setting changes and priority.

Xu et al. [114] designed a proof-of-work blockchain system, Healthchain, where health information is encrypted for fine-grained access control. Userchain and Docchain are two subblockchains, in Healthchain. AES, symmetric encryption, is used to encrypt IoT data. Data generated from doctor nodes are added to the blockchain by accounting nodes, which act as miners in Docchain, that are deployed by the consortium. The user can update it at any time by generating and transmitting a new key transaction if the IoT key or diagnosis key is compromised. In addition, 128-bit AES for symmetric encryption, SHA-256 for hash operation, and 1024-bit RSA for asymmetric encryption and signature are used. This work provides conditional security as it is assumed that private keys of both patients and doctors are secured and adversaries’ computing power is limited. This work involves a third-party audit to identify malicious transactions and malicious nodes as well.

Hospital’s private blockchain was designed by Liu et al. [115]. This work has a symptoms-matching mechanism that allows mutual authentication and creates a session key for their future communication about the illness of similar symptoms and it is implemented by using PBC and OpenSSL libraries. Proxy re-encryption technology is used to share data among doctors from different hospitals. Doctors will work as delegates to upload information and the hospital server will be responsible for block addition after the verification of information. This work involves public–private key-based encryption. For accessing the medical information of a patient, a doctor needs consent from patients, and secure communication for sharing encryption keys are not addressed.

Lin et al. [116] proposed a blockchain-based novel collective-reinforcement-learning algorithm to adaptively allocate resources based on the requirements of viewport rendering, block consensus, and content transmission. It considers an IoMT system where private medical services are embedded into a $360^{{}^{\circ}}$ omnidirectional video using virtual reality (VR) technology. To obtain service in the form of videos and feedbacks by virtual reality devices (VD), edge access points (EAPs) can reveice VR chunks from the application server to omnidirectional viewports like Liu et al. [115], or transmit them to the VD directly. In each time slot, block consensus is performed in five phases for authentication: (a) Request; (b) Pre-prepare; (c) Prepare; (d) Commit; and (e) Reply. MAC is used in the pre-prepare phase for authentication. In the commit phase, the blockchain controller in EAP signs the block and generates M-1 MACs for the non-primary nodes. One MAC of the Pre-prepare message and one signature of the block as well as signatures of the transactions included need to be verified by each non-primary node.

The blockchain-based solution [117] developed by Garg et al. allows for safe key management between implanted MDs and personal servers, as well as between personal servers and blockchain-managed cloud servers. The scheme is developed in eight stages and it uses three factors to improve security: (1) credentials that are stored in the device; (2) the password of user; (3) biometrics of user. It uses trusted authority only in the registration stage. After initial authentication between an MD and the personal server, both store secret key for future authentication. Sensors send data to the personal server, then, using the secret key personal server, sends data to the cloud server. Cloud servers use the Ripple Protocol Consensus Algorithm for verification and each cloud server uses an ECC-based private–public key pair.

Egala et al. designed a novel secured blockchain architecture that provides decentralized health records and a smart-contract based service automation system [118]. To improve security measures and maintain patient records anonymity, a decentralized selective ring-based access control (SRAC) mechanism is introduced along with ECC-based device authentication. It uses hybrid computing by logically combining edge and cloud computing. The main or global distributed data storage system (DDSS) is the combination of local DDSS networks which are created by using a sub-swarm key-generation process. A leader edge device in a random fashion is selected in the hospital to maintain a local cache of access control records, the public key pool, etc. This system uses smart contacts for writing business logic for decision making at the hybrid computing layer in the initial stage by patients or caretakers. An SRAC algorithm is used to validate and control access (Read/Write) with the help of a function. This framework also includes a gray list for unauthorized devices.

Table 3 shows a comparative analysis of blockchain-based IoMT security solutions.

### 6.8. NDN Based

Ullah et al. designed an authentication scheme using NDN in [119]. To reduce the key length, it used a hyperelliptic curve cryptosystem (HCC). It introduced a network manager to transfer public parameter sets and partial private keys between consumer and producer. It used a series of hashes (SHA-512) to produce signcrypted text and perform unsigncryption. In addition, Ref. [120] also used NDN to improve security. However, the framework suffers from a lot of map-to-map functions of bilinear pairing. In [121], Ullah et al. proposed a certificateless authentication framework using the NDN network. Here, HCC was used instead of ECC to provide similar-level security using low-size key length. It required low communication cost and communication overhead.

## 7. Conclusions

With the rapid advancement of interconnection between physical objects with the virtual world, IoT technology is able to gain the attention of attackers to identify flaws for gaining benefits. The research community and industry intellectuals are providing continuous efforts to make a robust system by eradicating vulnerabilities. Different kinds of solutions are being developed. To make the ecosystem safe and uniform, a common standard is necessary which will be maintained by manufacturers. To do this, the regulatory bodies such as the FDA and EU are making regulations and amending the standards to remove the loopholes in the system. Manufactures must have to follow the regulations to launch their products in the market. This paper shows the picture of the IoMT ecosystem, the functions of the IoMT system, and the threats on the network. This study performed a comprehensive analysis of the available security mechanisms for resource-constrained IoT devices to enhance security and maintain privacy. Furthermore, existing authentication schemes such as—ABE, ECC, MAC, ML, PUF, Blockchain—were considered. Moreover, NDN technology is incorporated here, which is under research for enhancing security. Scalability, memory size, computational resources, communication overhead, energy efficiency, and security are the main challenges of IoMT devices that need to be controlled before a successful integration takes place. In the future, the future of the IoMT in the era of the quantum system, 5G, and onward will be explored. Furthermore, the newly discovered security weaknesses such as those identified on June, 2022 for remote exploitation of cryptographic keys from Intel, AMD, and other microprocessors companies, will be investigated. In addition, the reason behind the vulnerabilities, and probable remedies, will also be inspected.

## Figures and Tables

**Table 1:** Comparative analysis of related works.

Survey	Citation	Year	Objective
A Comprehensive Survey on Attacks, Security Issues and Blockchain Solutions for IoT and IIoT	Sengupta et al. [17]	2019	Develop blockchain-based application-specific solutions for IoT and IIoT, and classify IoT attacks and responses.
From Pre-Quantum to Post-Quantum IoT Security: A Survey on Quantum-Resistant Cryptosystems for the Internet of Things	Fernández-Caramés et al. [19]	2020	Impacts and vulnerabilities of conventional and quantum security.
A Survey of Machine and Deep Learning Methods for Internet of Things (IoT) Security	Al-Garadi et al. [22]	2020	Hazards to IoT security and related solutions utilizing deep learning and machine learning.
A Survey on Physical Unclonable Function (PUF)-based Security Solutions for Internet of Things	Shamsoshoara et al. [18]	2020	PUF-based security mechanisms for IoT technology.
A Survey on the Integration of Blockchain With IoT to Enhance Performance and Eliminate Challenges	Sawadi et al. [25]	2021	Using blockchain technology, threats and risks are repelled.
A Survey on Security and Privacy Issues in Edge- Computing-Assisted Internet of Things	Alwarafy et al. [20]	2021	Utilizing edge computing to enhance data processing and intrusion resistance.
Public Blockchains for Resource-Constrained IoT Devices—A State-of-the-Art Survey	Khor et al. [26]	2021	Describe the benefits of blockchain technology and how resource-constrained devices could use it.
Machine Learning-Based Security Solutions for Healthcare: An Overview	Arora et al. [23]	2022	Healthcare security solutions using machine learning.
Utilization of mobile edge computing on the Internet of Medical Things: A survey	Awad et al. [27]	2022	Analyze the way to enhance quality and performance of IoMT using edge computing.
Machine Learning and Deep Learning Methods for Intrusion Detection Systems in IoMT: A survey	Rbah et al. [24]	2022	To make a defense system in IoMT using machine learning.
A survey on COVID-19 impact in the healthcare domain: worldwide market implementation, applications, security and privacy issues, challenges and future prospects	Shakeel et al. [21]	2022	Focused on healthcare systems, devices and different communication protocol rather than security systems.
**This Paper**	-	-	Ecosystem of healthcare, importance of IoMT in healthcare, EU and FDA regulations for healthcare with security issues, established security mechanisms and detailed discussion of different proposed security protocols.

**Table 2:** Comparative analysis of IoMT security schemes (centralized methods).

Author	Year	Objective	Technique Used	Type of Data	Framework	Pros	Cons
Deebak et al. [84]	2020	Data security and anonymity	PKI	Medical records	SSA	Solves Chiou et al.’s work [83]	Computational cost is high
Park et al. [85]	2020	To solve issues of MAKA scheme	PKI	Medical IoT data	LAKS-NVT	Does not require a server verification table	Traceable
Kumar et al. [87]	2020	Secure and efficient cloud-centric IoMT-enabled smart healthcare system	PKI	PHI file	EF-IDASC	Low energy consumption	DoS, reply attack
Limaye et al. [88]	2018	Facilitate research into new microarchitectures and optimizations	PKI	Healthcare data	HERMIT	Efficient processors for IoMT applications	Basic security
Lu et al. [89]	2020	TPM deployed in non-TPM protected embedded device via network	PKI	Sensor data	xTSeH	Does not discard request due to increased traffic	Security improvement required
Hsu et al. [90]	2020	Remove storing credentials and secure communication	PKI	eHealth data	UCSSO	No storage and central authority	Service could be interrupted
Chen et al. [91]	2021	Reduce energy consumption, achieve privacy and security	PKI & Chaotic map	Health data	-	Group authentication	Server impersonation
Li et al. [93]	2021	Reduce complexity and secure communication	PKI	Medical data	PSL-MAAKA	Lightweight scheme	Much time and storage required
Zhang et al. [94]	2020	Protect personal health records	ABE	PHR file	PHR sharing framework	Support offline and online	MITM, DoS, etc., security
Liu et al. [97]	2018	Enhance privacy preserving and efficient data structure	CP-ABE	Biomedical data	-	Server impersonation attack	Lot of storage and computation
Hwang et al. [95]	2020	Improve CP-ABE based scheme	CP-ABE	PHI file	-	Resolves key abuse problem	PHI leakage
Huang et al. [98]	2019	Protection from unauthorized entity	ECG	PHR file	-	Remove noise, light algorithm	No anonymous identity
Xu et al. [9]	2019	Secure data sharing	MAC	PHI file	-	Multi-keyword search	Device to gateway security
Siddiqi et al. [99]	2020	Security protocol for IMD ecosystem	MAC	Medical data	IMDfence	7% energy consumption	No user anonymity
Hahn et al. [96]	2020	Attack MAC-based scheme and countermeasure	Commitment (MAC)	Medical data	-	Low verification time	DoS, server impersonation
Li et al. [103]	2019	Enhance security of previous work	ECC	Medical data	3FUAP	Vulnerability and countermeasure	Computational cost
Almog- ren et al. [104]	2020	Fake node detection and deactivation	ECC	eHealth data	FTM	Double filter	Mainly focused on Sybil attack
Ying et al. [105]	2021	Secure communication	ECC	Medical data	-	Low computational time	High communication overhead
Liu et al. [106]	2021	Achieve data SNP preservation	ECC	EHR file	-	Major decryption on server side	Complex
Wang et al. [107]	2020	Ensure data privacy	Machine learning	Medical data	EPoSVM	Efficiency	Significant time required
Awan et al. [108]	2020	Maintains a robust network by predicting and eliminating malicious nodes	Supervised learning and ECC	Health data	NeuroTrust	Lightweight encryption	Needs focus on attacks
Ding et al. [109]	2020	To preserve the privacy or security of the patient	Deep learning	DeepEDN	Image	Fast	Needs robustness and server verification
Yanambaka et al. [111]	2019	Secure communication	PUF	Medical data	Pmsec	Lightweight	ML attack
Gope et al. [112]	2020	Secure and efficient authentication	PUF	Healthcare monitoring	-	Less computation at server	Two CRPs per transaction
Alladi et al. [78]	2020	To achieve physical security	PUF	Health data	HARCI	Low time in computation	Unstable CRP can cause failure

**Table 3:** Comparative analysis of decentralized IoMT security schemes (blockchain method).

Author	Year	Objective	Technique Used	Type of Data	Framework	Pros	Cons
Abdellatif et al. [113]	2021	Process large amounts of medical data	Blockchain	Medical data	MEdge-Chain	Remote monitoring, different actions for different data	Security is not focused
Xu et al. [114]	2019	Fine-grained access control	Blockchain (PoW)	Health data	Healthchain	User can update key if suspicious	Not suitable for high adversary
Liu et al. [115]	2019	Data sharing and privacy preservation	Blockchain (DPoS)	EHR file	-	Symptom matching communication	High overhead
Lin et al. [116]	2021	Task offloading and data processing to resist malicious attacks	Blockchain & MAC	VR video and feedback	-	Common view of similar patients	High computing capacity required
Garg et al. [117]	2020	Secure exchange of health-related confidential and private information	Blockchain & ECC	Health data	BAKMP	Dynamic node addition	Expensive computation
Egala et al. [118]	2021	Efficient secure exchange for decentralized network	Blockchain & ECC	EHR file	Fortified-Chain	Low energy, fast response	Ring tamper resistance instead of device

## Data Availability

Not applicable.
